# Biomimetic Scaffold with Aligned Microporosity Designed for Dentin Regeneration

**DOI:** 10.3389/fbioe.2016.00048

**Published:** 2016-06-08

**Authors:** Silvia Panseri, Monica Montesi, Samuele Maria Dozio, Elisa Savini, Anna Tampieri, Monica Sandri

**Affiliations:** ^1^Institute of Science and Technology for Ceramics, National Research Council, Faenza, Ravenna, Italy; ^2^Scuola Superiore “G. d’Annunzio”, University of Chieti-Pescara, Chieti, Italy

**Keywords:** mesenchymal stem cells, 3D cell culture, biomineralization, aligned porosity, dentin regeneration

## Abstract

Tooth loss is a common result of a variety of oral diseases due to physiological causes, trauma, genetic disorders, and aging and can lead to physical and mental suffering that markedly lowers the individual’s quality of life. Tooth is a complex organ that is composed of mineralized tissues and soft connective tissues. Dentin is the most voluminous tissue of the tooth and its formation (dentinogenesis) is a highly regulated process displaying several similarities with osteogenesis. In this study, gelatin, thermally denatured collagen, was used as a promising low-cost material to develop scaffolds for hard tissue engineering. We synthetized dentin-like scaffolds using gelatin biomineralized with magnesium-doped hydroxyapatite and blended it with alginate. With a controlled freeze-drying process and alginate cross-linking, it is possible to obtain scaffolds with microscopic aligned channels suitable for tissue engineering. 3D cell culture with mesenchymal stem cells showed the promising properties of the new scaffolds for tooth regeneration. In detail, the chemical–physical features of the scaffolds, mimicking those of natural tissue, facilitate the cell adhesion, and the porosity is suitable for long-term cell colonization and fine cell–material interactions.

## Introduction

Oral diseases represent a worldwide public health problem (Petersen, 2003). People usually lose their adult teeth because of several reasons ranging from lifestyle to genetic disorders, from aging to trauma (Chai and Slavkin, 2003).

Nowadays, prosthetic implants secured into the jawbone are the most common ways to overcome the problem of losing teeth. The earliest replacing lost teeth attempts have been dated back to the Aztecs, ancient Egyptian, Chinese, and Romans (Saini et al., 2015). Precious metals, ivory, or even other human teeth or piece of bone were used in their implants. Starting from twentieth century, several metals in addition to gold have been used, such as lead, iridium, tantalum, stainless steel, and cobalt alloy. Thanks to the extensive research work and advancement in material science, done in the last decades, new materials such as zirconia, roxolid, synthetic polymers, and surface-modified titanium implants are now available (Sykaras et al., 2000; Yuan et al., 2011; Sharma et al., 2014). However, all the current treatments are still far from a tissue regeneration approach.

A tooth is a complex organ that is composed of calcified tissues (dentin, enamel, and cementum) and soft connective tissues (the dental pulp and the periodontal ligaments) in which blood vessels and nerves are protected. Regenerating a whole tooth for clinical tooth replacement is now considered to be a challenging scientific goal (Du and Moradian-Oldak, 2006; Duraccio et al., 2015). This new paradigm requires scaffolds that balance temporary mechanical function with mass transport to aid biological delivery and tissue regeneration (Hollister, 2005).

The current use of calcium phosphate (Ca/P) materials (e.g., hydroxyapatite and β-tricalciumphosphate) similar to the mineral phase of the tooth (and bone) is a promising solution used in dental reconstructive surgery. Ca/P biomaterials have been widely tested for bone regeneration owing to their properties of resorption, biocompatibility, low immunogenicity, osteoconductivity, bone bonding, and similarity to mineralized tissues (Li et al., 2003; Sharma et al., 2014). Moreover, Ca/P granules have proved useful in dental tissue regeneration by providing suitable substrate for dental pulp stem cells (DPSC) growth and odontogenic differentiation (Nam et al., 2011). Also, natural polymers (e.g., collagen and hyaluronic acid) that represent the main structural components of extracellular matrix of several tissues have been extensively tested for dental regenerative medicine applications (Inuyama et al., 2010; Poursamar et al., 2015).

The combination of the above mentioned biomaterials provides biomimetic approaches suitable for dental tissue regeneration mixing the advantages of each individual material and minimizing their disadvantages. In this study, gelatin (Gel), denatured derivative of collagen, was biomineralized with magnesium-doped hydroxyapatite (MgHA) and blended with alginate (Alg) in order to obtain a biomimetic hybrid composite (MgHA-Gel/HA). Gelatin was chosen for its valuable features, such as non-immunogenicity, high biocompatibility, good biodegradability, and mostly for its low cost (Islam et al., 2015; Poursamar et al., 2015). Furthermore, cross-linking with Ca^2+^ ions and freeze casting processes were applied to shape the material in the form of porous 3D scaffolds with oriented microtubules resembling the architectural feature of dentin (Landi et al., 2008; Hunger et al., 2013).

A preliminary study with mesenchymal stem cells (MSCs) was performed in order to verify the suitability of the scaffold for cell colonization.

## Materials and Methods

### Scaffold Development

#### Development of Mineralized MgHA-Gel Hybrid Particles

A biomineralization procedure was carried out for the development of biomimetic hybrid particles containing ca. 80 wt% of MgHA nucleated on gelatin macromolecules.

First, gelatin (from pig skin, Italgelatine SpA, Italy) was dissolved at 40°C in water with a concentration of 5% w/v and kept under magnetic stirring for 1 h. The solution was then cooled at room temperature and 100 mL of H_3_PO_4_ (Sigma Aldrich, purity ≥85 wt%) 0.7M were added. After a complete homogenization, this solution was dropped in a Ca(OH)_2_ (Sigma Aldrich, purity ≥95 wt%) suspension (9.04 g in 500 mL of water) previously enriched with MgCl_2_·6H_2_O (Sigma Aldrich, purity ≥99 wt%) (1.19 g in 25 mL of water) to yield an MgHA/Gel composite material with a ratio of 80:20 wt%. The amount of MgCl_2_·6H_2_O was calculated to obtain an Mg/Ca molar ratio of 5%. The solutions were prepared with ultrapure water (0.22 μS, 25°C, MilliQ©, Millipore).

The acid mixture (pH = 3) was then slowly dropped to the basic suspension (pH = 12) containing Ca^2+^ and Mg^2+^ ions under vigorous and constant magnetic stirring. During the process, a slow decrease of pH up to neutrality was detected. At the end of the dripping, a white gel-like precipitate was observed and left to ripen in the mother liquor for 2 h.

The mineralized gelatin was then collected by centrifugation and cross-linked with 1,4-butanediol diglycidyl ether (BDDGE) setting up a BDDGE/Gelatin ratio of 1 wt% and incubating at 25°C for 2 days. The final product was then washed three times in 1 L of distilled water, dried into freeze-drier (5Pascal, Cinquepascal Srl, Italy), and sieved under 300 μm.

#### Development of MgHA-Gel/Alg Hybrid 3D Dentin-Like Scaffolds

The mineralized gelatin/alginate composite (MgHA-Gel/Alg) was produced dispersing and homogenizing in water the previously described MgHA-Gel particles with a concentration of 20 wt% and an alginate solution 8 wt% produced dissolving the sodium alginate in demineralized water at room temperature and completing the dissolution with 1 h of sonication. The alginate solution was then mixed with the MgHA-Gel dispersion with a ratio of 1:1, in order to obtain a MgHA-Gel:Alg ratio of 5:2. After 2 h of strong magnetic stirring, the hybrid gel was poured into 12-well multiwell plates and freeze-dryed (Cinquepascal srl, Italy) with a cycle: ↓ −50°C, ↑ 25°C. After freeze-drying, without removing the samples from the molds, 2 mL of 1M calcium chloride water solution was added to each well. The scaffolds were then washed from the excess of CaCl_2_ by placing them 10 times in fresh deionized water and freeze-dried again to preserve the cylindrical shape and the tubular porous structure.

As reference material for biological tests non-mineralized samples (Gel/Alg) were prepared with the same protocol of the mineralized one, but during the blending phase a 10 wt% gelatin solution was mixed with a 4 wt% alginate solution.

### Chemical–Physical and Morphological Characterization

Mineral phase composition and crystallinity degree were investigated by X-ray powder diffraction (XRD), and diffraction pattern was recorded by a Bruker AXS D8 Advance instrument in reflection mode (Cu-Kα radiation). The samples were ground through a cryomilling apparatus to obtain relatively uniform particle size powder. The composites were also examined by scanning electron microscopy (SEM) (Stereoscan 360, Leica, Cambridge, UK). ICP-OES quantitative analysis, by using an inductively coupled plasma atomic emission spectrometry (ICP-AES) (Liberty 200, Varian, Clayton South, VIC, Australia), was due to determine the content of Ca^2+^, PO_4_^3−^ and Mg^2+^ ions in the mineral phase. The samples were previously dissolved in nitric acid (Aldrich, 65 wt% pure). The obtained values were expressed in terms of (Mg + Ca)/P, Ca/P, and Mg/Ca mol%.

Thermogravimetric analysis (TGA) (Netzsch Gerätebau, STA449, Selb, Germany) was carried out to explore the thermal behavior of the composites and to assess the amount of mineral phase. This analysis was performed on specimens of about 20 mg and using a heating rate of 10°C min^−1^ up to 1000°C in air flow.

Scanning electron microscopy was used for the morphological characterization of scaffolds (ESEM Quanta 200, Fei). A cross-section and a longitudinal section per group was sputter coated with gold and observed by SEM.

The pore size of the scaffolds was evaluated by measuring the pores of five cross-sections per sample and three samples per group. Briefly, the scaffolds were soaked in PBS 1× (pH 7.4) for 24 h, then embedded in OCT overnight, and frozen with liquid nitrogen. 20 μm thickness cross-sections, obtained with microtome cryostat (5000 MC, Histo-line), were analyzed under bright field microscope (Ti-E microscope, Nikon) and the pore diameter measured with NIS Elements Imaging Software (3.22.1, Nikon). Results were expressed as mean ± SD.

### Cell Culture and Scaffold Seeding

Mouse MSCs (mMSCs) purchased from Lonza (Italy) were cultured in α-Modified Eagle’s Medium (α-MEM, Gibco), containing penicillin–streptomycin (100 U/mL–100 μg/mL) supplemented with 10% fetal bovine serum and kept at 37°C in an atmosphere of 5% CO_2_. Cells were detached from culture flasks by trypsinization and centrifuged; cell number and viability were assessed with trypan blue dye exclusion test. MgHA/Gel + Alg and MgGel + Alg scaffolds, 8.00 mm diameter and 4.00 mm high, were sterilized with ethanol and by UV irradiation (30 min). Samples were placed one per well in a 24-well plate and presoaked in culture medium for 72 h. Each scaffold was seeded by carefully dropping 20 μl of cell suspension (5.0 × 10^4^ cells) onto the upper scaffold surface, allowing cell attachment for 30 min, before addition into each well of 1.5 mL of cell culture medium supplemented with 100 μg/mL ascorbic acid, 25 mM β-glycerophosphate, and 10^−7^ M dexamethasone for cells differentiation. The medium was changed every 3 days. All cell handling procedures were performed in a sterile laminar flow hood. All cell culture incubation steps were performed at 37°C with 5% CO_2_.

### Cell Viability Assay

Live/dead assay kit (Invitrogen) was performed according to manufacturer’s instructions. Briefly, scaffolds were washed with 1× PBS for 5 min and incubated with calcein acetoxymethyl (calcein AM) 2 μM plus ethidium homodimer-1 (EthD-1) 4 μM for 15 min at 37°C in the dark. Samples were rinsed in 1× PBS, finely cut with a scalpel in order to examine also the internal surface. Images were acquired by an inverted Ti-E fluorescence microscope (Nikon). One sample per time point (1, 3, 7, and 14 days) was analyzed.

### Cell Morphology Analysis

Two samples for each time point were used for actin detection by fluorescence microscope and SEM analysis, respectively. Samples were finely cut with a scalpel in order to examine also the internal surface. In order to visualize actin filaments, samples were subjected to FITC-conjugated phalloidin staining followed by DAPI staining and visualized with an inverted Ti-E fluorescence microscope (Nikon). For SEM analysis, the samples were washed with 0.1M sodium cacodylate buffer pH 7.4 and fixed in 2.5% glutaraldehyde in 0.1M sodium cacodylate buffer pH 7.4 for 2 h at 4°C, washed in 0.1M sodium cacodylate buffer pH 7.4 and freeze-dried. Dehydrated samples were sputter coated with gold and observed using SEM (ESEM Quanta 200, Fei).

## Results and Discussion

Biomaterials for application in tissue engineering and regenerative medicine serve as three-dimensional templates and should provide appropriate microenvironments for cell adhesion, proliferation, differentiation, and neo tissue genesis. An advanced scaffold, therefore, may benefit from mimicking features of the native extracellular matrix.

In this study, a high reproducible anisotropic scaffold was designed and developed, mimicking chemical and tubular features of dentin tissue, and its behavior was analyzed with a preliminary 3D cell culture study.

### Preparation and Characterization of Nanostructured Hybrid MgHA-Gel Particles

The characteristics of the natural mineralized tissue are drastically different from those of artificial ones, and this is mainly the result of the absence of the peculiar self-organizing interaction between the synthetic mineral phase and the polymeric components.

In response to this problem, a biomimetic approach was followed and a biomineralization process was performed to develop a biohybrid material where the mineral and the organic phases reproduce the feature of the natural ones. Gelatin (Gel) was selected as a promising organic template for biomineralization process and low-cost material to develop 3D scaffolds (Kim et al., 2005).

**Figure 1:**
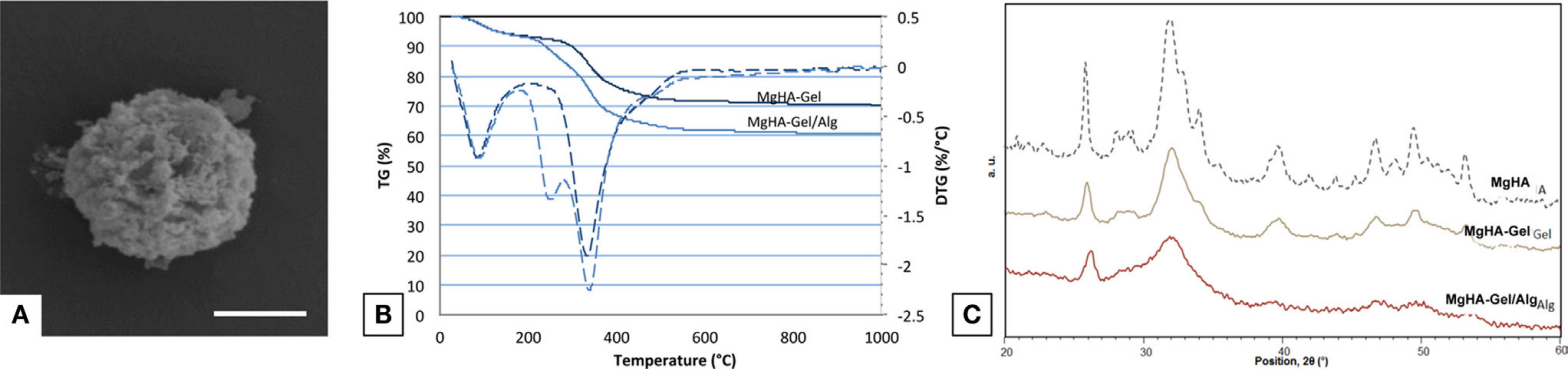
**Biomaterial characterization**. **(A)** Magnification of a MgHA-Gel hybrid particle; scale bar 2 μm. **(B)** TGA analyses of MgHA-Gel hybrid particles and MgHA-Gel/Alg scaffold. **(C)** XRD analyses of MgHA, MgHA-Gel and MgHA-Gel/Alg.

Hybrid MgHA-Gel microparticles (Figure 1A) were synthetized by means of a neutralization reaction allowing the nucleation of Mg-doped HA nanoparticles on gelatin molecules as organic template with a ratio of 70:30 wt% as revealed form TGA (Figure 1B). The mechanism occurring during the biomineralization process and the chemical–physical properties of the nucleated mineral phase have been largely investigated by Tampieri et al. (2004, 2008), demonstrating the possibility to synthetize highly biomimetic hybrid compounds suitable for hard tissue regeneration.

During the biomineralization process, the Ca^2+^ ions were able to interact with carboxylic functional groups of gelatin initiating the nucleation process of apatite nanocrystals on the polymeric matrix. Furthermore, during this mechanism, the self-assembling gelatin structure induces a structural confinement on the mineral phase promoting the nucleation of a quasi-amorphous MgHA reproducing the chemical and physical features of the natural apatite. The confinement effect exercised from the gelatin on apatite nanoparticles was clearly demonstrated from the broadened diffraction pattern of XRD spectra collected on the MgHA-Gel hybrid micro particles (Figure 1C). XRD spectra of MgHA-Gel reveal a low-crystalline apatite with a crystallinity extent much lower than the MgHA prepared in the same conditions but without the organic template (Figure 1C) and confirm the inhibition of the mineral phase crystallization due to its interaction with the organic template. The crystallinity of the mineral phase plays a very important role because it can influence its biological activity (toward cells), the kinetic of biodegradation, and its physical and mechanical properties.

The chemical mimesis of natural dentinal tissues was enhanced by the incorporation of Mg^2+^ ions in the apatite phase (MgHA) during the biomineralization process; furthermore, as previously demonstrated, Mg^2+^ ions are responsible to hinder the excessive crystallization of apatite particles with the ensuing formation of a highly biomimetic mineral phase (Bertinetti et al., 2006; Tampieri et al., 2011). ICP analysis confirms the presence of Mg in the hybrid particles at a level of 70% with respect to the one nominally introduced as reagent. The molar ratio (Mg + Ca)/P is 1.69 (Table 1), while Ca/P ratio is 1.64 lower than the theoretical one and confirming the replacement of Ca with Mg.

**Table 1:** **ICP analyses of MgHA-Gel and MgHA-Gel/Alg composites**.

	(Mg + Ca)/P (mol)	Ca/P(mol)	Mg/Ca (mol)	P (wt%)	Ca (wt%)	Mg (wt%)
MgHA-Gel	1.69 ± 0.02	1.64 ± 0.03	3.5 ± 0.3	14.4 ± 0.3	30.4 ± 0.4	0.60 ± 0.02
MgHA-Gel/Alg	1.92 ± 0.02	1.87 ± 0.05	3.0 ± 0.2	14.6 ± 0.3	34.7 ± 0.5	0.59 ± 0.03

### Preparation and Characterization of MgHA-Gel/Alg Scaffolds

**Figure 2:**
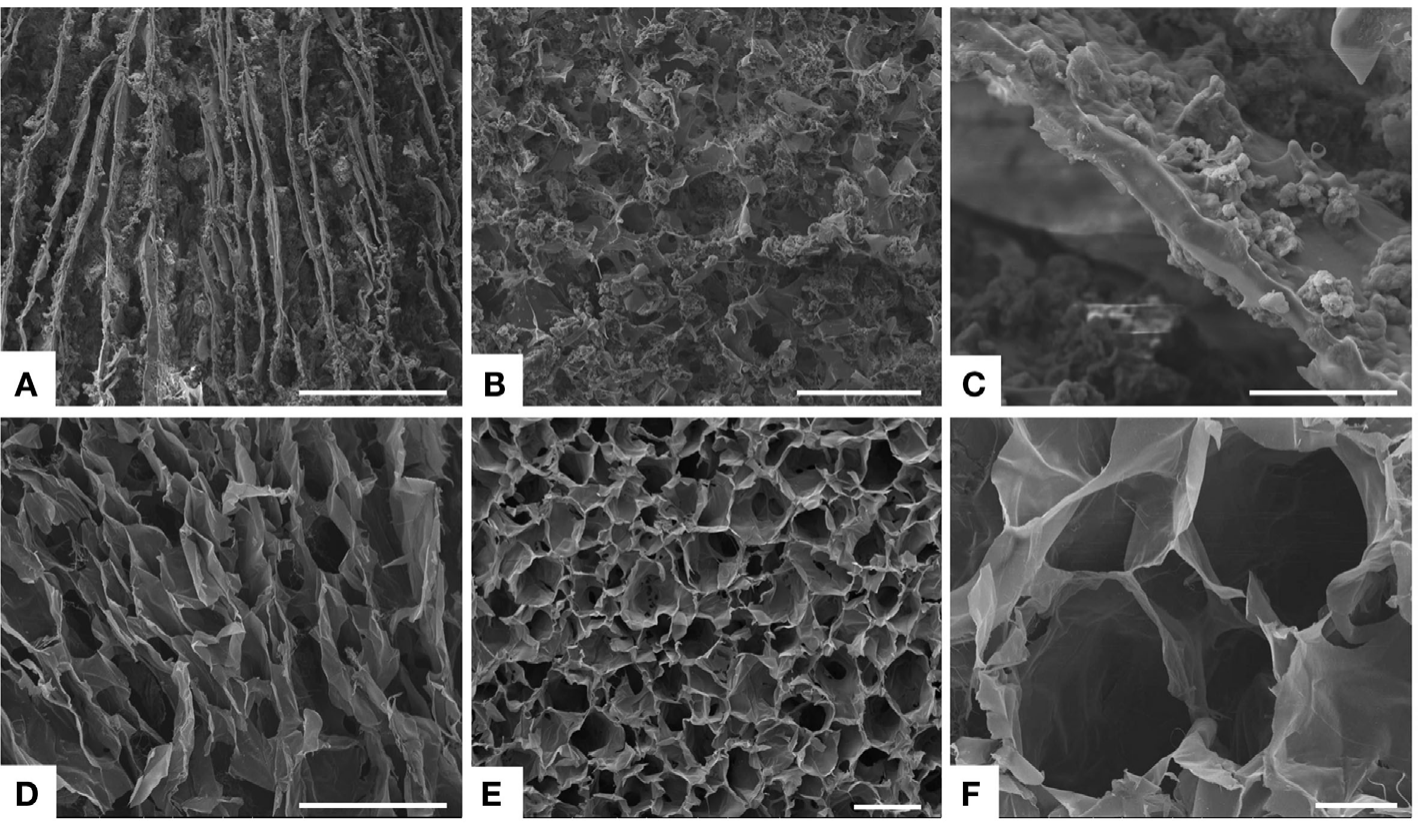
**SEM analysis**. **(A)** Longitudinal section and **(B)** transversal section of the freeze-dried hybrid scaffold MgHA-Gel/Alg. In figure **(C)** a details of the mineralized gelatin micro-particles (MgHA-Gel) merged in the alginate matrix. **(D)** Longitudinal section and **(E,F)** transversal section of the freeze-dried polymeric scaffold Gel/Alg as reference material. Scale bars: **(A,D)** 500 μm; **(B,E)** 200 μm; **(C,F)** 50 μm.

The biomimetic MgHA-Gel particles have been subsequently used to develop nanostructured 3D scaffolds mimicking dentin tubules by embedding them into an alginate matrix. Alginate (Alg), a natural polysaccharide extracted from brown seaweed, was selected because of its well-assessed physical and biological properties, such as biocompatibility, low immunogenicity, gelation capacity, and aptitude to generate channel-like structures (Zmora et al., 2002). Moreover, its strong interaction with Ca^2+^ ions allows the formation of stable blends with apatitic particles and their chemical stabilization by means of intermolecular cross-linking process. Alg amount was kept as lower as possible in order to get a stable 3D channel-like structure without reduction of the total mineral content. To achieve a 3D anisotropic and channel-like structure, the hybrid blend MgHA-Gel/Alg was freeze-dried applying a rapid freezing and reaching a low cooling temperature (−50°C), suitable conditions to produce thin and lamellar pores (Figure 2A) (Landi et al., 2008). In our experience, applying freeze-drying to blended HA/Alg or biomineralized HA/Alg aqueous composites results in the formation of a softer and faster degrading porous network (Tampieri et al., 2005; Dittrich et al., 2007). Thus, after the first freeze-drying process, the scaffolds were treated with Ca^2+^ ions in water solution, then washed, and freeze-dried again. This procedure generate intramolecular linking between alginate molecules (Lee and Mooney, 2012), thus providing the strengthening and the chemical stabilization of the channel-like porous structure in physiological-like conditions.

The proposed scaffolds showed open porous and interconnected networks and suitable porosity with aligned microtubules to resemble the dentin structures and to favor the initial cell colonization (Figure 2) (Loh and Choong, 2013). Detailed analysis of pores dimension was performed on 20-μm-thick scaffold cross-sections. Data in Table 2 indicated that MgHA-Gel/Alg scaffold had pores smaller than Gel/Alg scaffold (170.1 ± 7.3 versus 253.8 ± 14.9), even if both scaffolds had the majority of the pores >150 μm (MgHA-Gel/Alg ≈ 61.4%; Gel/Alg ≈ 78.5%).

**Table 2:** **Scaffold porosity analysis (mean ± SD)**.

	HA/Gel + Alg	Gel + Alg
Pores (μm)	170.1 ± 7.3	253.8 ± 14.9
Pores <150 μm	125.9 ± 4.8 (≈38.6%)	90.9 ± 6.6 (≈21.5%)
Pores >150 μm	199.1 ± 8.1 (≈61.4%)	298.8 ± 15.4 (≈78.5%)

Scanning electron microscopy morphological analysis of cross and longitudinal sections (Figures 2A,B) showed the channel-like porous structure. With regard to the hybrid particle (MgHA-Gel) distribution, at higher magnification (Figure 2C), it can be seen that the hybrid particles are well integrated and uniformly distributed in the alginate matrix. SEM analyses performed on the polymeric reference material presented analogous porous structure in terms of channel dimension and orientation (Figures 2D–F).

Inductively coupled plasma analysis confirms the preservation of the mineral phase during the blending process. The (Mg + Ca)/P (1.92) and Ca/P (1.87) molar ratio in MgHA-Gel/Alg showed a slight increase with respect to the MgHA-Gel (Table 1) because of the presence of Ca^2+^ ions cross-linking the alginate molecules, whereas the Mg wt% (0.59) content was unchanged.

Moreover, XRD analysis confirmed that the blending process does not modify the structure and the crystallinity of the inorganic phase (Figure 1C). The XRD analysis performed on MgHA-Gel/Alg composite has evidenced the presence of broadened peaks ascribed to the inorganic (MgHA) phase that shows the same features of the starting MgHA-Gel particles (Figure 1C).

Figure 1B shows the TGA and its first derivative (DTG) profiles of MgHA-Gel and MgHA-Gel/Alg samples. In both cases, the weight loss centered at 75°C is due to the evaporation of adsorbed structural water and that in the range 180–550°C present in both MgHA-Gel and MgHA-Gel/Alg samples is due to the degradation of polymeric components, alginate and gelatin. DTG curve of MgHA-Gel shows a single degradation peak while that of MgHA-Gel/Alg scaffold shows a double degradation peek demonstrating the presence of Alg beside Gel. As previously reported (Chang et al., 2003; Soares et al., 2004; Tampieri et al., 2005), Alg and Gel completely degrade within 1000°C;, thus, the final residual mass of 60 wt% corresponds to the effective content of mineral phase (MgHA) in the dentin-like scaffold that is quite similar to the mineral content of natural dentin tissue (Low et al., 2008).

### 3D Cell Culture on MgHA-Gel/Alg Scaffolds

**Figure 3:**
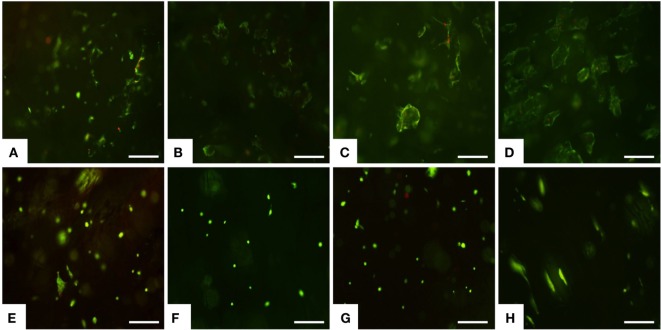
**Cell viability was analyzed by the Live/Dead assay**. Calcein AM stains for live cells in green, EthD-1 stains for dead cells in red. **(A–D)** MgHA-Gel/Alg and **(E–H)** Gel/Alg scaffolds. Live/Dead assay was performed at day 1 **(A,E)**, day 3 **(B,F)**, day 7 **(C,G)** and day 14 **(D,H)**. Scale bars 200 μm.

**Figure 4:**
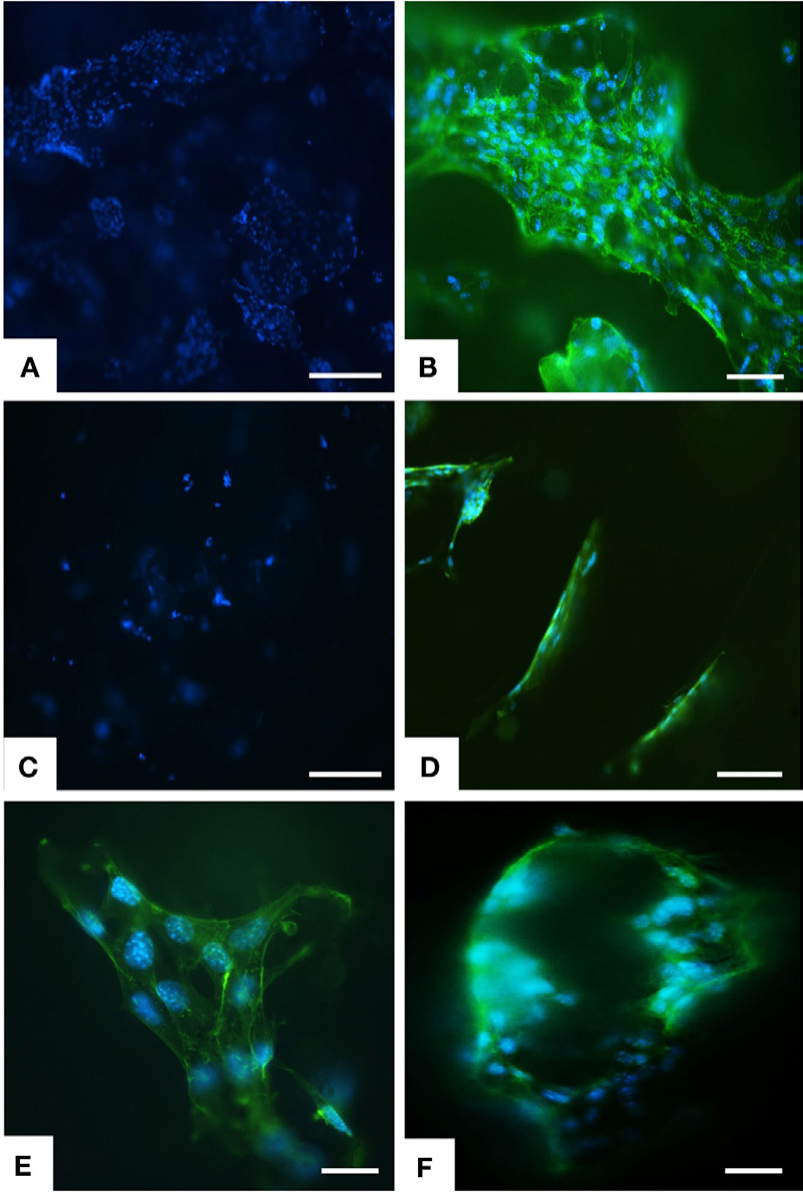
**Analysis of scaffold colonization and cell morphology by DAPI and phalloidin staining**. After 7 days several cells were seen on MgHA-Gel/Alg scaffold **(A)** respect to Gel/Alg scaffold **(C)**. MSCs were spread with good morphology and firmly attached to MgHA-Gel/Alg **(B)** respect to Gel/Alg scaffold **(D)**. Details of cells grown on the inner scaffold surface and on the pore’s edge respectively in **(E)** and **(F)**. Phalloidin in green stains for actin filaments and DAPI in blue stains for cell nuclei. Scale bars **(A–D)** 100 μm; **(E)** 20 μm; **(F)** 50 μm.

**Figure 5:**
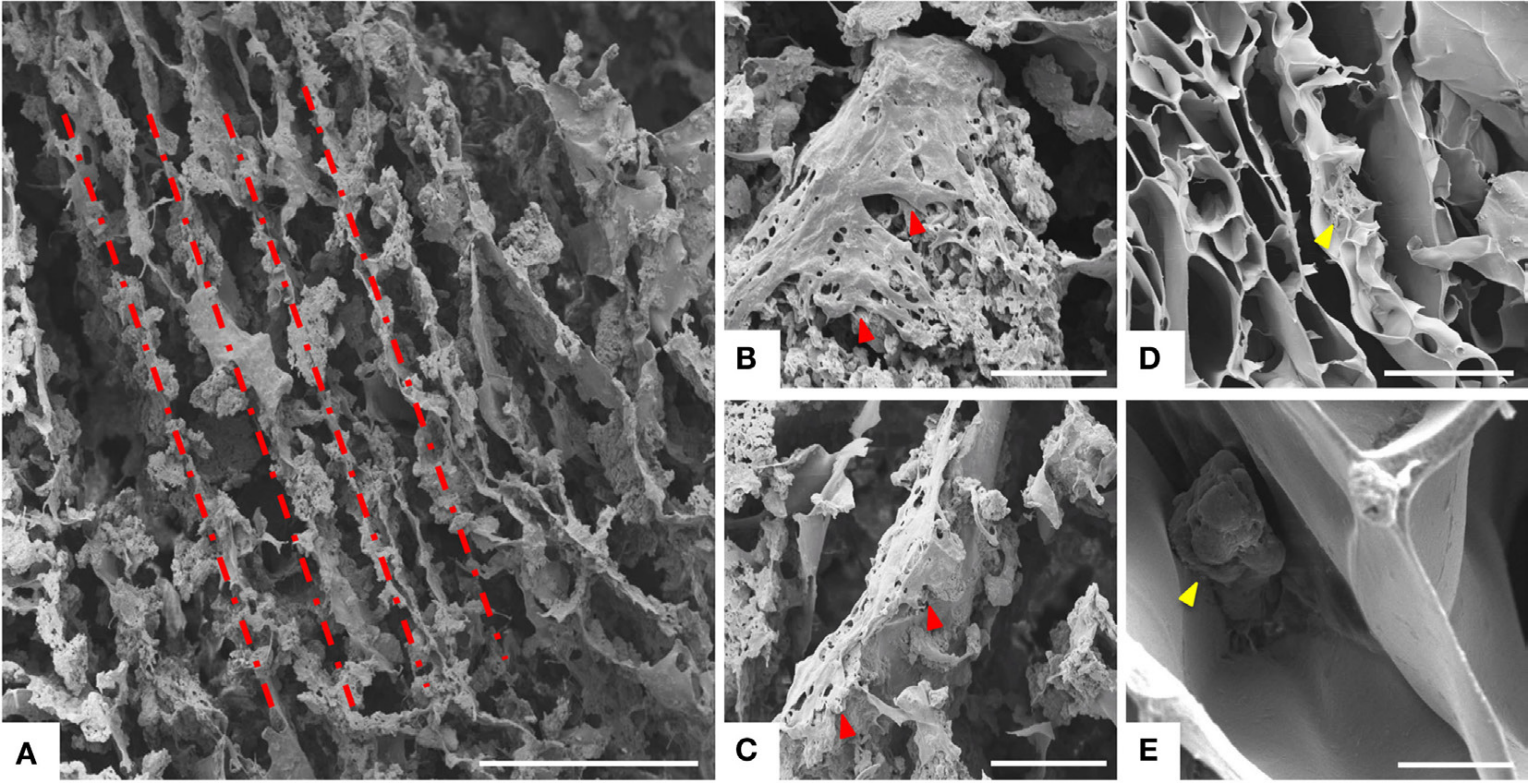
**Analysis of cell morphology assessed by SEM**. MSCs grown on MgHA-Gel/Alg scaffold **(A–C)** and on Gel/Alg scaffold **(D,E)** respectively. Dashed line highlights the aligned porosity. Red arrows indicated some MSCs on MgHA-Gel/Alg scaffold; yellow arrows indicated some MSCs on Gel/Alg scaffold. Scale bars: **(A)** 500 μm; **(B,C)** 100 μm; **(D)** 300 μm; **(E)** 20 μm.

The preliminary biological study was performed to verify the suitability of novel low-cost mineralized scaffold in cell colonization and cell adhesion. MSCs, originally identified in the bone marrow, have been isolated from numerous tissues including dental pulp, and they have high proliferative and multipotent capacity leading to differentiated cells under the guidance of various cues and niches (Gnecchi and Melo, 2009; Huang et al., 2009; Ledesma-Martinez et al., 2016). For these reasons, MSCs were used and seeded on the upper scaffold surface, and the cell viability was tested after 1 day up to 14 days by live/dead assay based on the simultaneous determination of live and dead cells with two probes, Calcein and EthD-1, which measure recognized parameters of cell viability: intracellular esterase activity and plasma membrane integrity, respectively (Papadopoulos et al., 1994). A very high ratio of viable MSCs was seen on MgHA-Gel/Alg at each time points (Figures 3A–D). Concerning the Gel/Alg, even though very few dead cells were detected (Figures 3E–H), it was clear that the total number of cells was lower compared to MgHA-Gel/Alg group. It is well known that cellular behavior is greatly influenced by surface properties, including hydrophilicity, texture, morphology, and roughness (Hu et al., 2011). In detail, rough surfaces were found to enhance initial cell adhesion resulting in better bone/dental fixation than smooth surfaces (Zan et al., 2016). This *in vitro* study supports this hypothesis: the roughness of the mineralized scaffold due to the presence of HA strongly enhance the adhesion of MSCs as shown also in Figures 4 and 5.

Greater initial cell attachment to rough MgHA-Gel/Alg surfaces was followed by strong scaffold colonization. Seven days after the cell seeding, whole mineralized scaffold colonization was seen with a significant difference with respect to the Gel/Alg scaffold (Figures 4A,C). Moreover, the cytoskeletal structure, detected by phalloidin that stains actin filaments (i.e., essential element in maintaining and modulating cellular morphology) and SEM analysis, showed well spread MSCs cultured on MgHA-Gel/Alg scaffold with respect to Gel/Alg scaffold (Figures 4 and 5). Moreover, the nuclear morphological qualitative analysis of MSCs seeded on the MgHA-Gel/Alg samples showed their native morphology and no abnormal alterations were detected (e.g., nuclear fragmentation and chromatin condensation) (Figure 4E). The aligned porosity allowed scaffold colonization as proved by cross-sections where cells were detected on the circumference of the pores (Figures 3D and 4F) and by longitudinal sections where MSCs are grown along the pores’ edges (Figure 5A).

## Conclusion

This preliminary study indicated the low-cost biomineralized gelatin blended with alginate scaffold as promising tool for 3D cell culture in dental regeneration. The aligned porosity synergistically with the rough surfaces, due to the presence of HA, strongly enhanced the initial cell adhesion and consequent whole scaffold colonization. Although the effect of HA of MSCs differentiation is already well known, further experiments will be essential to prove that mineralized gelatin scaffolds positively influence dentinogenesis.

## Author Contributions

SP, MM, MS, and AT conceived and designed the project. ES and SD synthesized and characterized the biomaterial. SP, SD, and MM performed the biological study. SP, MM, and MS analyzed the data. SP and MS wrote the manuscript. All authors read and approved the final manuscript.

## Conflict of Interest Statement

The authors declare that the research was conducted in the absence of any commercial or financial relationships that could be construed as a potential conflict of interest.
